# Mental health in the Austrian general population during COVID-19: Cross-sectional study on the association with sociodemographic factors

**DOI:** 10.3389/fpsyt.2022.943303

**Published:** 2022-11-24

**Authors:** Elke Humer, Yvonne Schaffler, Andrea Jesser, Thomas Probst, Christoph Pieh

**Affiliations:** Department of Psychosomatic Medicine and Psychotherapy, University for Continuing Education Krems, Krems, Austria

**Keywords:** COVID-19, depression, anxiety, insomnia, alcohol abuse, eating disorders, stress, sociodemographic status

## Abstract

**Introduction:**

The impact of the Coronavirus disease (COVID-19) pandemic and the associated governmental restrictions on mental health have been reported in different countries. This cross-sectional study evaluated mental health during the COVID-19 pandemic in Austria and the association with sociodemographic factors (i.e., age, sex, education, income, employment status, partnership status, and migration background).

**Methods:**

A representative sample (*N* = 1,031) of the Austrian general population was surveyed online end of April 2022. Indicators of mental health were depression (PHQ-9), anxiety (GAD-7), insomnia (ISI), alcohol abuse (CAGE), eating disorders (SCOFF), and stress (PSS-10).

**Results:**

1,031 participants completed the online survey (50.3% women; mean age: 45.6 ± 17.23 years). Cut-offs for clinically relevant depression were exceeded by 28%. 16% scored above the cut-off for clinically relevant anxiety symptoms, 15% for clinical insomnia, 18% for alcohol abuse, 26% for eating disorders, and 65% for moderate to high stress. Comparisons with another cross-sectional representative Austrian sample recruited during the first weeks of the COVID-19 pandemic in Austria (April 2020) revealed increases in depression (from 21 to 28%) but no significant changes in anxiety, insomnia, and moderate to high stress. Multivariable logistic regression showed the strongest associations of mental health indicators with age, income, and sex. Increasing age and income were associated with lower odds of mental health symptoms. Being female compared to male increased the odds of depressive symptoms while decreasing the odds of alcohol abuse.

**Discussion:**

The COVID-19 crisis seems particularly stressful for younger adults (<35 years) and people with low income (<€2,000 net household income per month). Policymakers need to consider the high social and economic costs of lockdowns and think of optimal intervention methods for mental disorders among young and low-income individuals.

## Introduction

The outbreak of the Coronavirus disease (COVID-19) pandemic in early 2020 disrupted lives across all countries and communities and negatively affected physical health, mental health, and the economy. Several studies highlight that the pandemic and associated measures to combat the spreading of the virus negatively affected mental health, causing increases in the prevalence of depression, anxiety, insomnia, substance abuse, eating disorders, and stress (1–3). Fears of illness, reduced social contacts, and financial concerns have been suggested to be important factors underlying the detrimental effects of the pandemic on mental health (4). Loneliness resulting from self-isolation during the pandemic was defined as a typical mental health concern in the era of COVID-19 (5). It was found to be significantly positively correlated with anxiety, depression, and high stress (6). Two years after the emergence of the pandemic, most countries lifted most protective measures; however, whether the reduction in daily confirmed COVID-19 cases and relaxation of protective measures are associated with improved mental health remains unknown so far.

**FIGURE 1:**
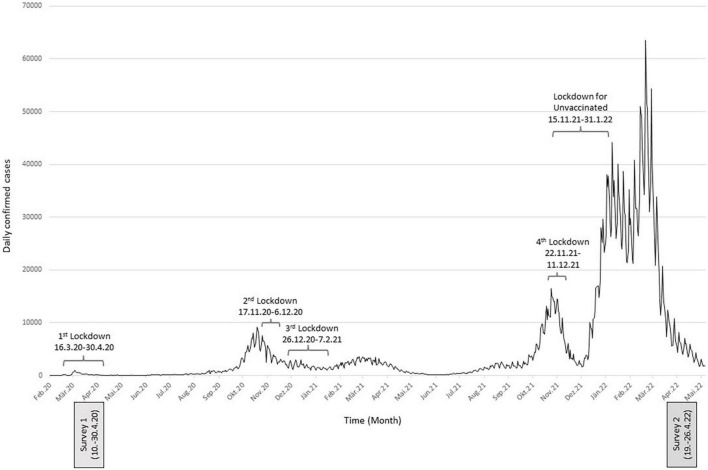
Daily confirmed COVID-19 cases in Austria, lockdown measures in place, and sampling times.

In Austria, the first COVID-19 cases were detected at the end of February 2020, followed by the first nationwide strict COVID-19 lockdown from the middle of March 2020 until the end of April 2020 as depicted in Figure 1. A representative survey conducted in April 2020–after the first 4 weeks of lockdown–in the general population (*n* = 1,005) revealed higher mental health symptoms (21% depression, 19% anxiety, 16% insomnia) compared to pre-pandemic data (7). The strict lockdown was followed by low daily confirmed COVID-19 cases and relaxed protective measures until the summer of 2020. Re-evaluations of mental health 6 weeks (8), and 6 months (9) after the end of the lockdown *via* longitudinal studies, revealed no improvement in mental health. As daily confirmed COVID-19 cases and hospitalization rates increased in autumn/winter 2020 (the second wave of COVID-19 infections in Austria), further lockdown measures were introduced from the middle of November 2020 until the beginning of February 2021 (second and third strict nationwide lockdowns which were only interrupted in the mid of December to allow for Christmas shopping and limited family gatherings around the holidays). During this time, a cross-sectional survey investigating the mental health of the general population (*n* = 1,505) was conducted on a representative sample of the Austrian general population. This survey even observed a further increase in the prevalence of mental health disorders [26% depression, 23% anxiety, 18% insomnia (10)]. After the openings in February 2021, the third wave of infections (the Beta variant) reached Austria, accompanied by further regional strict lockdown measures in the eastern part of Austria. In the spring/summer of 2021, daily confirmed COVID-19 cases declined, vaccination rates increased, and a series of easing of COVID-19 restrictions came into effect. In late summer 2021, the fourth wave of infections (the Delta variant) affected Austria. In the mid of November 2021, new measures were introduced that increasingly restricted various areas of public life, such as shopping beyond basic needs, gastronomy, hairdressers, etc., for unvaccinated people. Subsequently, a fourth strict nationwide lockdown was in place starting in late November 2021. While the general lockdown ended in the middle of December, the lockdown for unvaccinated people remained in place until the end of January 2022. In late December 2021, the Omicron variant spread, and the fifth wave of infections emerged in Austria (11, 12). Although no strict lockdown occurred during the fifth wave of infections, several protective measures remained in place until the end of March 2022. In April 2022, COVID-19 restrictions were strongly lifted. FFP2 masks were only mandatory in essential shops (i.e., supermarkets, pharmacies, and banks), public transport and taxis, and hospitals and nursing homes. The “3-G” rule (vaccinated, recovered, or tested) was lifted for restaurants, bars, and events and only applied when entering Austria. During this period of lifted protective measures, we conducted a cross-sectional (“Survey 2” in Figure 1), representative online survey to assess the mental health status of the general population after 2 years of repeated restriction and relaxation measures. Therefore, the first aim of this study was to explore the prevalence of mental disorders in the Austrian general population in April 2022 and compare results with the first cross-sectional survey conducted in April 2020 (“Survey 1” in Figure 1).

Disasters such as a pandemic reveal essential information concerning the creation of particular patterns of social (13), structural (14), or syndemic (15–17) vulnerability. They interact with socioeconomic, cultural, and contextual determinants of health, which contribute to poorer physical health and accumulation of social disadvantages (18) and poorer mental health (19, 20). The COVID-19 pandemic as a global crisis is currently exacerbating the vulnerabilities of people in difficult life situations and social, economic, and political divisions in society are becoming more apparent than before (21). However, it remains unclear along which exact pathways vulnerability affects a population’s mental health and to which degree vulnerable groups are affected as the pandemic progresses.

As the conditions of daily life largely determine the vulnerability of individuals, households, and social groups (13), a second aim was to examine whether the risks of mental disorders are associated with sociodemographic status. Previous studies have proposed several sociodemographic risk factors for impaired mental health, including younger age, female sex, low education, unemployment, migration background, low income, and living as a single (7, 10, 22–24). Due to inequitable social orders, these groups tend to have limited social and economic resources or share other structural vulnerabilities (14). As several sociodemographic factors are not independent of each other (e.g., lower income in people with lower education), the aim of our study was not only to examine the association between a single sociodemographic variable and mental health but also to investigate the independent contribution of each sociodemographic variable in predicting the prevalence of mental health disorders by adjusting for the other sociodemographic variables.

## Materials and methods

An online survey was conducted between 19 and 26 April 2022. A representative sample of the Austrian general population was recruited from a pre-existing online access panel provided by Marketagent.com online research GmbH (Baden, Austria; certified under ISO 20252). Participants had to be at least 14 years old, reside in Austria, and have access to the internet and sufficient German skills to participate in the study. Marketagent has about 130,000 registered panelists in Austria (25). Using quota sampling, respondents were selected and invited based on quotas for the following key demographics: age, sex, age × sex, region, and educational level. The sociodemographic characteristics of the study sample (*N* = 1,031) are summarized in Table 1. Supplementary Table 1 summarizes the categories used for quota sampling, showing the intended quota (based on data from the Austrian Federal Statistical Office) vs. the final quotas reached in the current survey.

**TABLE 1:** Study sample characteristics (*n* = 1,031).

	*N*	%
**Sex**		
Male	512	49.7
Female	519	50.3
**Age (years old)**		
14–24	140	13.6
25–34	176	17.1
35–44	182	17.7
45–54	171	16.6
55–64	193	18.7
≥65	169	16.4
**Region**		
Vienna	225	21.8
Upper Austria	168	16.3
Lower Austria	195	18.9
Carinthia	61	5.9
Styria	150	14.5
Tyrol	89	8.6
Salzburg	65	6.3
Burgenland	34	3.3
Vorarlberg	44	4.3
**Education**		
No school education	9	0.9
Secondary school	212	20.6
Apprenticeship	350	33.9
Vocational secondary school	169	16.4
High school	169	16.4
University	122	11.8
**Migration background**		
Yes	140	13.6
No	891	86.4
**Work situation**		
In employment	591	57.3
Unemployed	199	19.3
Retired	241	23.4
**Net household income**		
<€ 1,000	134	13.0
€ 1,000–€ 2,000	292	28.3
€ 2,001–€ 3,000	254	24.6
€ 3,001–€ 4,000	179	17.4
>€ 4,000	172	16.7
**Partnership status**		
Single	322	31.2
Living in partnership	709	68.8

Migration background was defined as whether both parents were born abroad (second-generation immigrants) or participants themselves were born abroad (first-generation immigrants).

Two years earlier, a representative sample of the Austrian general population (*n* = 1,005) was surveyed, using the same measures for depressive symptoms, anxiety, insomnia, and stress as in the present study. These measures were selected since they are validated in German and commonly used in the research literature to assess mental health disorders (1, 26, 27). Results from the first survey are already published [Pieh et al. (7)], and data on the prevalence of mental disorders are used in the present study for comparative purposes only.

This study was conducted following the Declaration of Helsinki and approved by the Ethics Committee of the University for Continuing Education Krems, Austria (Ethical number: EK GZ 26/2018–2021). All participants gave electronic informed consent to participate and complete the questionnaires.

### Measures

#### Sociodemographic status

To assess the sociodemographic status, the following seven variables were evaluated: Participants were asked about their sex, age, highest education (no school; secondary school; apprenticeship; vocational secondary school; higher secondary school; university), net household income per month (<€ 1000; € 1001–€ 2000; € 2001–€ 3000; € 3001–€ 4000; >€ 4000), work situation (employed; unemployed; retired), migration status (whether they or both parents were born abroad or not); partnership status (single; partnership). Due to the low number of participants with no school education, the categories “no school” and “secondary school” were combined for further analyses.

#### Depressive symptoms (PHQ-9)

The depression module of the Patient Health Questionnaire (PHQ-9) was used to assess depressive symptoms (28). The PHQ-9 comprises nine self-rating items on a four-point scale from 0 to 3, yielding a total score from 0 to 27, with higher values indicating more severe depressive symptoms. A cut-off point of at least 10 points is defined as moderate, clinically relevant depression in adults (29), while a cut-off of ≥11 indicates moderate depression in adolescents (30). Thus, a cut-off of ≥11 was applied for participants aged between 14 and 17, while a cut-off of ≥10 was used for participants aged 18 or older. Cronbach’s alpha was α = 0.89 in the present sample.

#### Anxiety (GAD-7)

The Generalized Anxiety Disorder 7 scale (GAD-7) was applied to measure anxiety symptoms (31). The seven self-rating items measure anxiety on a four-point scale from 0 to 3, with a cut-off score of 10 defining moderate, clinically relevant anxiety in adults (32). The suggested cut-off of ≥11 for moderate anxiety [Mossman et al. (33)] was applied for adolescents (14–17 years). Cronbach’s alpha was α = 0.91 in the present sample.

#### Insomnia (ISI)

Sleep quality was measured with the Insomnia Severity Index (ISI). The seven items of the ISI measure sleep quality and insomnia on a five-point scale from 0 to 4, with a cut-off score of 15 defining moderate, clinically relevant insomnia (34). Cronbach’s alpha was α = 0.89 in the present sample.

#### Alcohol problems (CAGE)

Problematic alcohol use was assessed with the Cut down, Annoyance, Guilty, Eye-opener (CAGE) screening interview (35). The CAGE comprises four yes/no questions targeting signs of alcoholism (questions about Cutting down, Annoyance with criticism, Guilty feelings, and Eye-openers). A cut-off of two questions answered with “yes” indicates problematic alcohol use (36). Cronbach’s alpha was α = 0.67 in the present sample.

#### Eating disorders (SCOFF)

Eating disorders were assessed with the Sick, Control, One, Fat, Food (SCOFF) screening interview (37). The acronym SCOFF describes five yes/no key screening questions for eating disorders: “Sick, Control, One stone, Fat, Food.” A cut-off of two questions answered with “yes” indicates eating disorders (38). Cronbach’s alpha was α = 0.53 in the present sample.

#### Perceived stress (PSS-10)

Perceived stress levels were measured with the Perceived Stress Scale (PSS-10). The ten items of the PSS-10 measure stress on a five-point scale from 0 to 4, with a cut-off score of 14 defining moderate stress levels (39). Cronbach’s alpha was α = 0.85 in the present sample.

### Statistical analyses

Descriptive statistics were conducted to describe sociodemographic characteristics. Chi-squared tests were applied to assess differences in the prevalence of clinically relevant depression, anxiety, insomnia, and stress between the current cross-sectional study and the cross-sectional study conducted 2 years earlier (7).

Univariate associations of mental health outcomes (clinically relevant depression, anxiety, insomnia, eating disorders, alcohol misuse, and stress) and sociodemographic variables (age, sex, education, income, work situation, migration background, and partnership status) were analyzed by chi-squared tests. *P*-values of less than 0.05 were considered statistically significant (2-sided tests). As a measure of association, Phi (φ) was used as the effect size equivalent for the chi-squared statistics.

Using multivariable logistic regression, we adjusted the data for sociodemographic variables (age, sex, education, income, work situation, migration background, and partnership status). Adjusted odds ratios (OR) and their 95% confidence intervals (CIs) were estimated to assess the statistical uncertainty. All statistical analyses were performed using SPSS version 26 (IBM Corp., Armonk, NY, USA).

## Results

### Mental health symptoms in April 2022 and comparison to April 2020

In April 2022, cut-offs for clinically relevant depression (PHQ-9 ≥10 points for participants ≥18 years and ≥11 points for participants aged 14–17 years) were exceeded by 28.3%. 16.1% scored above the GAD-7 cut-off ≥ 10 points (adults) and ≥11 points (adolescents aged 14–17 years) for clinically relevant anxiety symptoms, 14.5% above the cut-off ≥ 15 points (ISI) for clinical insomnia, 17.9% above the cut-off ≥ 2 points (CAGE) for alcohol abuse, 26.1% above the cut-off ≥ 2 for eating disorders (SCOFF), and 64.8% above the cut-off ≥ 14 (PSS-10) for moderate stress.

Data of all participants aged 18 years or older (*n* = 1,011) were compared to the data of a representative sample of the Austrian general population aged 18 years or older (*n* = 1,005), which were recruited during the first weeks of the COVID-19 pandemic in Austria (April 2020). Comparisons revealed higher levels of depression from 21.0% in April 2020 to 28.0% in April 2022 (*p* < 0.001). No differences were observed for anxiety (19.0% in 2020 vs. 15.9% in 2022; *p* = 0.069), insomnia (15.7% in 2020 vs. 14.3% in 2022; *p* = 0.386), and moderate/high stress (61.6% in 2020 vs. 64.4% in 2022; *p* = 0.193).

### Association of sex with mental health problems

**FIGURE 2:**
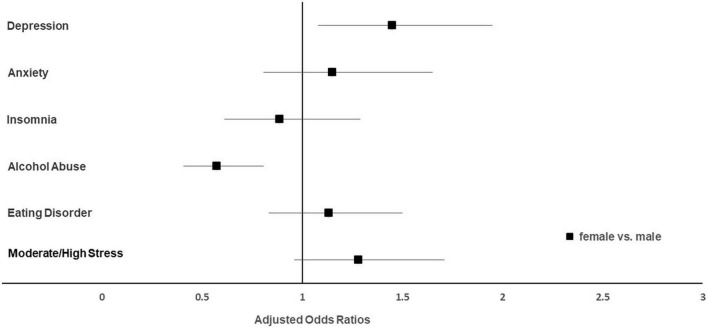
Adjusted odds ratios stratified by gender with the category “male” being the reference.

Univariate analyses revealed a higher prevalence of depression and moderate to high stress levels in women compared to men (*p* < 0.05), while the opposite was observed for alcohol abuse (*p* = 0.003; Supplementary Table 2). When adjusting for all investigated sociodemographic variables, the associations of sex with depression and alcohol abuse remained significant. As depicted in Figure 2 women, compared to men, were more likely to experience clinically relevant depression (aOR 1.45), but less likely to exceed the cut-off for alcohol abuse (aOR 0.57).

### Association of age with mental health problems

According to univariate analyses (Supplementary Table 3), the prevalence of all investigated mental health problems decreased with increasing age. Multivariable logistic regression analyses mainly confirmed these findings (Table 2), with lower odds for depression (aORs 0.13–0.49) and alcohol abuse (aORs 0.20–0.42) in participants older than 44 years compared to those between 14 and 24 years. The odds for anxiety disorders (aORs 0.14–0.45) and eating disorders (aORs 0.26–0.46) decreased in participants older than 54 compared to participants aged between 14 and 24. Moderate to stress levels were less prevalent in individuals older than 34 (aORs 0.25–0.52) compared to the youngest age group (14–24).

**TABLE 2:** Adjusted odds ratios (ORs) stratified by age group with the age category “14–24 years” being the reference.

	25–34 years vs.			35–44 years vs.			45–54 years vs.			55–64 years vs.			65+ years vs.		
	14–24 years			14–24 years			14–24 years			14–24 years			14–24 years		
Variable	OR	95% CI		OR	95% CI		OR	95% CI		OR	95% CI		OR	95% CI	
Depression	0.79	0.48	1.28	0.73	0.45	1.20	**0.49**	**0.29**	**0.82**	**0.36**	**0.21**	**0.63**	**0.13**	**0.06**	**0.30**
Anxiety	1.08	0.61	1.92	1.05	0.59	1.87	0.59	0.32	1.12	**0.45**	**0.23**	**0.88**	**0.14**	**0.05**	**0.40**
Insomnia	1.26	0.66	2.43	1.64	0.86	3.10	1.45	0.75	2.80	0.90	0.44	1.83	0.37	0.13	1.03
Alcohol abuse	0.68	0.39	1.19	0.86	0.50	1.48	**0.42**	**0.23**	**0.78**	**0.34**	**0.17**	**0.65**	**0.20**	**0.08**	**0.50**
Eating disorder	0.63	0.38	1.04	0.68	0.41	1.12	0.70	0.42	1.17	**0.46**	**0.27**	**0.81**	**0.26**	**0.12**	**0.56**
Moderate/High stress	0.69	0.37	1.29	**0.52**	**0.28**	**0.97**	**0.45**	**0.24**	**0.83**	**0.27**	**0.14**	**0.49**	**0.25**	**0.11**	**0.53**

Significant associations are marked in bold. The multivariable regression model was adjusted for sex, income, work, migration background, education, and partnership status. 14–24 years was the reference group. Nagelkerke R^2^: depression: 0.16; anxiety: 0.12; insomnia: 0.13; alcohol abuse: 0.09; eating disorder: 0.08; moderate/high stress: 0.22.

### Association of net household income with mental health problems

**FIGURE 3:**
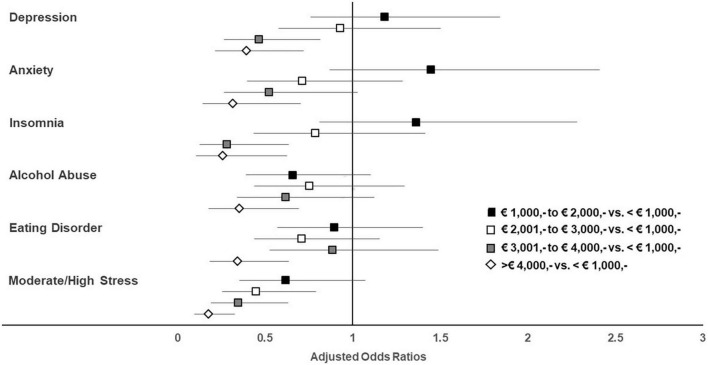
Adjusted odds ratios stratified by income with the category “<€ 1,000,-” being the reference.

The net household income showed strong associations with all mental health indicators, not only in univariable (Supplementary Table 4) but also in multivariable analyses (Figure 3). After adjusting for all investigated variables addressing the sociodemographic status, lower likelihoods for depression (aORs 0.40–0.47) and insomnia (aORs 0.26–0.29) were observed in adolescents with a net household income exceeding € 3,000 vs. <€ 1,000. The odds for anxiety disorders (aOR 0.32), alcohol abuse (aOR 0.35), and eating disorders (aOR 0.35) decreased in participants with a net household income exceeding € 4,000 vs. <€ 1,000. Individuals with a net household income exceeding € 2,000 vs. <€ 1,000 were less likely to experience moderate to high stress levels (aORs: 0.18–0.45).

### Association of employment status with mental health problems

The job situation showed significant associations with mental health indicators when analyzed independently of other sociodemographic variables (Supplementary Table 5). More specifically, the highest prevalences of depression, anxiety, insomnia, and moderate to high stress levels were observed in unemployed participants, while the lowest were observed in retired individuals (*p* < 0.05). For alcohol abuse, the highest prevalences were observed in employed individuals and the lowest in retired (*p* = 0.038). After taking all investigated variables addressing the sociodemographic status into account, only the association of the employment situation with the perceived stress level remained significant (Table 3). Retired individuals were less likely to experience moderate to high stress levels than employed individuals (aOR 0.58).

**TABLE 3:** Adjusted odds ratios (ORs) stratified by job situation with being employed as reference category.

	Unemployed vs.			Retired vs.		
	employed			employed		
Variable	OR	95% CI		OR	95% CI	
Depression	1.23	0.85	1.77	1.22	0.69	2.18
Anxiety	0.86	0.55	1.34	1.44	0.73	2.86
Insomnia	1.31	0.84	2.04	1.40	0.71	2.76
Alcohol abuse	0.64	0.41	1.02	1.42	0.72	2.76
Eating disorder	0.90	0.62	1.33	1.31	0.75	2.32
Moderate/High stress	1.40	0.90	2.15	**0.58**	**0.35**	**0.98**

Significant associations are marked in bold. The multivariable regression model was adjusted for age, sex, income, migration background, education, and partnership status. Nagelkerke R^2^: depression: 0.16; anxiety: 0.12; insomnia: 0.13; alcohol abuse: 0.09; eating disorder: 0.08; moderate/high stress: 0.22.

### Association of education with mental health problems

In univariate analyses, the highest education was associated with specific mental health indicators (Supplementary Table 6). Prevalences of depression, insomnia, eating disorders, and moderate to high stress levels were lowest in participants who attended university and highest in those with no school education or those who attended secondary school. No associations between education and mental health indicators were found when all other sociodemographic variables were included in the statistical analyses (Table 4).

**TABLE 4:** Adjusted odds ratios (ORs) stratified by education with the education category “no or secondary school” being the reference.

	Apprenticeship vs.			Vocational secondary school vs.			High school vs.			University vs.		
	no/Secondary school			no/Secondary school			no/Secondary school			no/Secondary school		
Variable	OR	95% CI		OR	95% CI		OR	95%	CI	OR	95%	CI
Depression	1.12	0.76	1.66	1.05	0.65	1.71	1.07	0.67	1.71	0.66	0.356	1.217
Anxiety	0.87	0.55	1.38	0.86	0.48	1.53	0.70	0.39	1.25	0.84	0.416	1.697
Insomnia	0.93	0.58	1.48	1.03	0.58	1.82	0.67	0.36	1.25	0.47	0.197	1.139
Alcohol abuse	0.74	0.46	1.16	0.87	0.50	1.52	0.86	0.51	1.47	0.94	0.499	1.751
Eating disorder	0.85	0.58	1.26	0.82	0.51	1.32	0.90	0.56	1.43	0.63	0.346	1.139
Moderate/High stress	0.71	0.47	1.08	0.97	0.60	1.59	0.78	0.47	1.29	0.80	0.469	1.376

The multivariable regression model was adjusted for age, sex, income, work, migration background, and partnership status. Nagelkerke R^2^: depression: 0.16; anxiety: 0.12; insomnia: 0.13; alcohol abuse: 0.09; eating disorder: 0.08; moderate/high stress: 0.22.

### Association of migration background with mental health problems

Chi-squared tests revealed a higher prevalence of alcohol abuse, eating disorders, and moderate to high stress levels in participants with migration backgrounds than in those without (*p* < 0.05; Supplementary Table 7). Only the association with eating disorders and moderate/high stress levels remained significant in the multivariable analyses, indicating 1.50 (eating disorders) and 1.65 (stress) higher adjusted odds for eating disorders in participants with migration backgrounds than those without (Table 5).

**TABLE 5:** Adjusted odds ratios (ORs) stratified by migration with having no migration background as the reference category.

	Migration background yes vs. no		
Variable	OR	95% CI	
Depression	1.13	0.75	1.69
Anxiety	0.86	0.52	1.42
Insomnia	1.03	0.62	1.70
Alcohol abuse	1.42	0.92	2.21
Eating disorder	**1.50**	**1.01**	**2.22**
Moderate/High stress	**1.65**	**1.04**	**2.61**

Significant associations are marked in bold. The multivariable regression model was adjusted for age, sex, income, work, education, and partnership status. Nagelkerke R^2^: depression: 0.16; anxiety: 0.12; insomnia: 0.13; alcohol abuse: 0.09; eating disorder: 0.08; moderate/high stress: 0.22.

### Association of partnership status with mental health problems

Compared to those living as single, participants living in a partnership had a lower prevalence of depression, anxiety, insomnia, and moderate/high stress levels (*p* < 0.05; Supplementary Table 8). In the multivariable analyses, no associations of partnership status with all investigated mental health indicators were found (Table 6).

**TABLE 6:** Adjusted odds ratios (ORs) stratified by partnership status with being single as the reference category.

	Partnership vs. single		
Variable	OR	95% CI	
Depression	0.79	0.57	1.11
Anxiety	0.88	0.59	1.30
Insomnia	0.87	0.58	1.31
Alcohol abuse	1.14	0.77	1.70
Eating disorder	1.02	0.73	1.43
Moderate/High stress	0.90	0.63	1.27

The multivariable regression model was adjusted for age, sex, income, work, migration background, and education. Nagelkerke R^2^: depression: 0.16; anxiety: 0.12; insomnia: 0.13; alcohol abuse: 0.09; eating disorder: 0.08; moderate/high stress: 0.22.

## Discussion

This cross-sectional study, with participants being representative of the Austrian population, shows that mental health problems remained at a high level even in spring 2022, when only minimal restrictions were in place. The proportion of individuals exceeding cut-offs for clinically relevant depression even increased compared to the first year of the pandemic. Moreover, after adjusting for potential confounders, we found that younger age and low income are the main risk factors for mental health disorders.

Overall, when compared to other countries, Austria had rather strict COVID-19 policies, including several strict lockdown measures for the general population during the first year of the pandemic and additional measures for unvaccinated individuals during the second year of the pandemic. Results of the current study suggest that repeated lockdowns negatively impact the human psyche in the long term and that already disadvantaged groups were more affected. A recent meta-analysis on the effects of lockdowns suggests that they had no or only marginal public health benefits, but went along with vast socio-economic costs (40). Our findings confirm that lockdowns have had vast socio-economic costs, as they suggest that the socially vulnerable are disproportionately affected by mental health distress. As a consequence, the earning capacity of the low-income group could be particularly limited, thus widening the gap between the rich and the poor and potentially further decreasing economic growth (41). Also, the effect of the differentiated treatment of vaccinated and unvaccinated individuals in Austria (i.e., the lockdown for unvaccinated people in winter 2021/2022), on public health is unknown, given the limited effect of vaccination on SARS-CoV-2 transmission (42). A potential effect of the vaccination status on mental health indicators was analyzed in the present study to reveal a potential detrimental effect of being unvaccinated on mental health, as unvaccinated people stayed longer in confinement. However, as none of the analyzed mental health indicators differed between vaccinated and unvaccinated individuals, the results of the current study do not provide evidence for differences in mental health in vaccinated vs. unvaccinated individuals.

When interpreting the results, it should be considered that the past 2 years have been dominated by several crises, not only the COVID-19 pandemic. The Russia-Ukraine war, inflation, money issues, the climate change are other major global issues that impact daily lives and likely mental health. Considering the recent and future inflation, the observed relationship between household income and risk for mental health problems is highly relevant.

The findings on the strong association of age with mental health are in line with previous studies, indicating worse mental health in younger adult groups (<35 years), especially in adolescents (7, 10, 43). In the current study, the youngest age group (14–24 years) showed 2.70–7.73 higher adjusted odds for exceeding the cut-offs of clinically relevant mental health symptoms. Overall, increasing age was strongly associated with a decreased risk of mental disorders. This might seem contradictory at first glance, especially since older adults (60+ years) experience an increased risk of dying from COVID-19, suggesting more significant worry about COVID-19 and thus worse mental health (44). However, a growing body of evidence suggests that the oldest adults handle the COVID-19 situation better than the younger ones (23, 24). This has been commonly explained by the specific biographical challenges and biopsychosocial changes of adolescence and early adulthood (45). Young people increasingly detach themselves from their parents and the nuclear family. Reorientation to peers of the same age facilitates young people’s development into independent adults. It allows them to develop a sense of social self-identity while building stronger bonds with their peer group (46). As an essential context for peer interaction and the acquisition of knowledge and personal maturity, the school contributes significantly to the development of adolescent identity and interpersonal relationships (47). However, during the COVID-19 pandemic, students were repeatedly taught *via* distance learning over a longer period. School closures and curfews resulted in the loss of critical social contact with classmates and friends. Daily routines changed overnight as distance learning was introduced (48). Similarly, young adults may have experienced the transition to home office and measures to limit social contact and public life. Experiences with friends, shared leisure activities, attending social events, and traveling do have an identity-building role in this age group as well.

Another explanation for the great strain on adolescents and young adults are the uncertainties that currently result for young people in the field of education and professional life (49, 50). For those who are still in education or transitioning to working life, the pandemic has led to an interruption of education and employment biographies (51). As the pandemic progresses and economic problems increase, studies reveal growing fears among young people regarding learning outcomes and prospects (52, 53). Unemployment rates for young people under 25 in Austria were almost twice as high as in the general population in February 2022 (54).

Another suggested reason for young people’s vulnerability to mental health problems is the better capacity of older adults to regulate their emotions and manage stress than younger adults (44, 55).

The study results at hand support the general notion that mental disorders affect more socially disadvantaged people (19, 20). Education is a crucial indicator of socioeconomic status, as people with low education are increasingly left behind (56). Although lower education was associated with a higher mental health burden in the univariate analysis, the association vanished when other indicators of socioeconomic status were considered simultaneously. This is likely due to the confounding role of income with education, as people with low education have fewer prospects for secure, financially stable employment (56). Household income showed the strongest association with mental health indicators among socioeconomic status indicators. Participants in the lowest income category (net household income lower than € 1,000 per month) were at 2.53–5.66 increased odds of all investigated mental health disorders. Our study supports the notion of the association between income and increased risk for incident mental disorders (57). Several presumed mechanisms underlie the association between low income and mental health problems. For example, low-income populations found it harder to adhere to non-pharmacological interventions, to get tested, isolate, and obtain treatment when necessary (58), and they have been exposed to more chronic stressors linked to deleterious genetic and hormonal changes, increasing the risk of developing or exacerbating mental health issues (59, 60). Low socioeconomic status has also been associated with risky health behaviors, including smoking and problematic alcohol use (61, 62). Poverty is also associated with decreased capacity for seeking appropriate mental and physical healthcare (57, 63).

Social inequalities not only sculpt the distribution of emerging health problems and the course of illness in those affected (64) but also magnify existing inequalities (58). More disadvantage, in turn, means higher exposure to stressors. Also, considering the economic problems both in the aftermath of the pandemic and caused by the current Russia-Ukraine war in Europe, a trend toward worsening mental health is expected for low-income groups. Worse mental health, in turn, will hurt economic income opportunities, so the spiral continues to turn. Recommendations have been made to protect vulnerable populations, reduce health inequities (58), and strengthen governmental mental health responses (65). Austria has a well-developed social safety net and compulsory health insurance compared to many other countries. However, while statutory health insurance fully covers acute psychiatric care, reimbursement rates in psychotherapy are low. One recommendation is that governments should take immediate action to strengthen their mental health systems and services to meet the increased demands for mental health and psychosocial support. They should see the COVID-19 pandemic as an opportunity to reinforce their country’s mental health system in preparation for future emergencies and to build it better and fairer (65). There is also room for improvement in the right of diverse population groups to have a say in decision-making in handling a collective crisis. Much has been undertaken that favored protection of the older population from infection with COVID-19, while little emphasis has been placed on protecting the mental health of younger individuals. Knowledge of the needs of disadvantaged populations could be better used to develop solutions that meet the needs of all populations. Existing non-governmental organizations can reach and protect low-income and other vulnerable groups in ways that governments cannot, as they have the trust of these people in ways that governments often do not. They should receive more funding to design and implement solutions that meet the needs of the full range of populations (58).

This study has several limitations. First, no causal conclusion can be drawn due to the study’s cross-sectional design. The high prevalence of mental health indicators, as well as their association with sociodemographic factors cannot be solely linked to the COVID-19 pandemic, as the study was conducted during a period dominated by other crises, e.g., connected to the Ukraine–Russia conflict. This intra-European conflict brought many Ukrainian refugees to Austria. Tensions between Russia and Euro-American countries led to expensive food, energy, and an atmosphere of uncertainty. Besides the pandemic, the war within Europe was another dominant and emotionally discussed theme in Austrian newspapers at the time of the survey. Given a multilevel crisis of such magnitude, the general population was likely dealing with diverse and significant issues, such as fear of illness, unemployment, economic recession, and worrying sociopolitical developments (66).

Second, mental health indicators were based solely on self-reports. For valid statements regarding the diagnosis of mental disorders, a structured clinical interview would have been required.

Third, although the sample was representative of age, sex, education, and region, only age and sex were interlocked. However, by adjusting for multiple sociodemographic variables simultaneously in the multivariable regression analyses, we aimed to balance this limitation as far as possible.

Fourth, the moderate and low internal consistencies of the CAGE (0.67) and SCOFF (0.53) scales raise concerns about their reliability. However, the internal consistency analysis is likely influenced by the binary answer layout (yes/no) and might have also been affected by the low number of items (CAGE: 4 items; SCOFF: 5 items).

Fifth, further important variables, such as previous COVID-19 infection as well as the respective severity of a previous infection, professional occupation, and living situation among others should be taken into account in future studies.

## Conclusion

The emergence of COVID-19 has drawn attention to the inequalities and injustices in mental health outcomes experienced by groups that are more likely than others to be disadvantaged and marginalized. The findings of this study contribute to an understanding that social determinants of mental health, in particular young age and low income, are associated with inequalities in the incidence of depressive symptoms, anxiety, sleep disorders, alcohol problems, eating disorders, and perceived stress in the context of the COVID-19 pandemic. The present study confirms the high societal costs of lockdowns and underscores the need for targeted mental health interventions to detect and treat mental health problems among people younger than 35 and individuals with a household income of less than € 2,000 per month.

## Data availability statement

The raw data supporting the conclusions of this article will be made available by the authors, without undue reservation.

## Ethics statement

The studies involving human participants were reviewed and approved by University for Continuing Education Krems, Krems, Austria. Written informed consent to participate in this study was provided by the participants’ legal guardian/next of kin.

## Author contributions

EH and CP: conceptualization and methodology. EH: formal analysis, investigation, and data curation. EH, YS, and AJ: writing—original draft preparation. TP and CP: writing—review and editing. All authors read and agreed to the published version of the manuscript.
