# Supplementary material for: Mental health in the Austrian general population during COVID-19: Cross-sectional study on the association with sociodemographic factors
Source: Front Psychiatry. 2022 Nov 24;13:943303. doi: 10.3389/fpsyt.2022.943303 (PMC9729349; doi:10.3389/fpsyt.2022.943303)
Supplement: Supplementary file 1 [file Data_Sheet_1.docx]

Supplementary Material

**Supplementary Table 1** Quota sampling categories in the current sample vs. the representative Austrian general population (based on data from the Austrian Federal Statistical Office)

|  | Sample % | Representative % | % Difference |
| --- | --- | --- | --- |
| **Sex** |  |  |  |
| Male | 49.7 | 49.3 | 0.4 |
| Female | 50.3 | 50.7 | -0.4 |
| **Age** |  |  |  |
| 14-24 | 13.6 | 13.7 | -0.1 |
| 25-34 | 17.1 | 16.1 | 1 |
| 35-44 | 17.7 | 15.8 | 1.9 |
| 45-54 | 16.6 | 17.6 | -1 |
| 55-64 | 18.7 | 17.2 | 1.5 |
| ≥65 | 16.4 | 19.5 | -3.1 |
| **Region** |  |  |  |
| Vienna | 21.8 | 21.5 | 0.3 |
| Upper Austria | 16.3 | 16.8 | -0.5 |
| Lower Austria | 18.9 | 18.9 | 0 |
| Carinthia | 5.9 | 6.3 | -0.4 |
| Styria | 14.5 | 14.0 | 0.5 |
| Tyrol | 8.6 | 8.5 | 0.1 |
| Salzburg | 6.3 | 6.3 | 0 |
| Burgenland | 3.3 | 3.3 | 0 |
| Vorarlberg | 4.3 | 4.5 | -0.2 |
| **Education** |  |  |  |
| No or secondary school | 21.5 | 24.8 | -3.3 |
| Apprenticeship | 33.9 | 31.1 | 2.8 |
| Vocational secondary school | 16.4 | 13.8 | 2.6 |
| High School | 16.4 | 17.3 | -0.9 |
| University | 11.8 | 13.0 | -1.2 |

**Supplementary Table 2** Proportion of participants exceeding the cut-off scores for moderate depression/anxiety/insomnia/alcohol abuse/eating disorders and stress by sex (n = 1,031)

|  | |  | Sex | | *P* | φ |
| --- | --- | --- | --- | --- | --- | --- |
| Variable | |  | male  (n = 512) | female  (n = 519) |  |  |
| Depression | % (n) | | 23.8% (122) | 32.8% (170) | 0.001 | 0.099 |
| Anxiety | % (n) | | 14.1% (72) | 18.1% (94) | 0.077 | 0.077 |
| Insomnia | % (n) | | 13.9% (71) | 15.2% (79) | 0.537 | 0.029 |
| Alcohol Abuse | % (n) | | 21.5% (110) | 14.5% (75) | 0.003 | -0.092 |
| Eating Disorder | % (n) | | 24.2% (124) | 27.9% (145) | 0.174 | 0.042 |
| Moderate/High Stress | % (n) | | 60.2% (308) | 69.4% (360) | 0.002 | 0.096 |

**Supplementary Table 3** Proportion of participants exceeding the cut-off scores for moderate depression/anxiety/insomnia/alcohol abuse/eating disorders and stress by age (n = 1,031)

|  | |  | |  | |  | | Age | |  | |  | |  | | *P* | | φ | |
| --- | --- | --- | --- | --- | --- | --- | --- | --- | --- | --- | --- | --- | --- | --- | --- | --- | --- | --- | --- |
| Variable | |  | | 14-24  (n = 140) | | 25-34  (n = 176) | | 35-44  (n = 182) | | 45-54  (n = 171) | | 55-64  (n = 193) | | 65+ (n = 169) | |  | |  | |
| Depression | % (n) | | 45.7% (64) | | 35.2% (62) | | 33.0% (60) | | 26.3% (45) | | 22.8% (44) | | 10.1% (17) | | <0.001 | | 0.237 | |  |
| Anxiety | % (n) | | 22.1% (31) | | 21.6% (38) | | 21.4% (39) | | 14.0% (24) | | 13.0% (25) | | 5.3% (9) | | <0.001 | | 0.165 | |  |
| Insomnia | % (n) | | 15.0% (21) | | 15.3% (27) | | 18.7% (34) | | 17.5% (30) | | 14.0% (27) | | 6.5% (11) | | 0.027 | | 0.111 | |  |
| Alcohol Abuse | % (n) | | 27.1% (38) | | 20.5% (36) | | 25.3% (46) | | 14.0% (24) | | 13.0% (25) | | 9.5% (16) | | <0.001 | | 0.167 | |  |
| Eating Disorder | % (n) | | 38.6% (54) | | 26.1% (46) | | 28.0% (51) | | 29.2% (50) | | 22.3% (43) | | 14.8% (25) | | <0.001 | | 0.156 | |  |
| Moderate/High Stress | % (n) | | 85.7% (120) | | 76.1% (134) | | 70.3% (128) | | 67.3% (115) | | 53.4% (103) | | 40.2% (68) | | <0.001 | | 0.304 | |  |

**Supplementary Table 4** Proportion of participants exceeding the cut-off scores for moderate depression/anxiety/insomnia/alcohol abuse/eating disorders and stress by income (n = 1,031)

|  | |  | |  |  | Income |  |  | *P* | φ |
| --- | --- | --- | --- | --- | --- | --- | --- | --- | --- | --- |
| Variable | |  | | < € 1,000  (n = 134) | € 1,000,- to € 2,000,- (n = 292) | € 2,001,- to € 3,000,- (n = 254) | € 3,001,- to € 4,000,-  (n = 179) | > € 4,000  (n = 172) |  |  |
| Depression | % (n) | | 38.1% (51) | | 38.4% (112) | 28.7% (73) | 17.9% (32) | 14.0% (24) | <0.001 | 0.304 |
| Anxiety | % (n) | | 20.9% (28) | | 25.3% (74) | 13.4% (34) | 10.6% (19) | 6.4% (11) | <0.001 | 0.215 |
| Insomnia | % (n) | | 20.1% (27) | | 23.6% (69) | 14.2% (36) | 5.6% (10) | 4.7% (8) | <0.001 | 0.192 |
| Alcohol Abuse | % (n) | | 25.4% (34) | | 16.8% (49) | 19.7% (50) | 17.9% (32) | 11.6% (20) | 0.033 | 0.216 |
| Eating Disorder | % (n) | | 35.1% (47) | | 30.1% (88) | 24.0% (61) | 28.5% (51) | 12.8% (22) | <0.001 | 0.101 |
| Moderate/High Stress | % (n) | | 83.6% (112) | | 73.3% (214) | 63.8% (162) | 59.2% (106) | 43.0% (74) | <0.001 | 0.156 |

**Supplementary Table 5** Proportion of participants exceeding the cut-off scores for moderate depression/anxiety/insomnia/alcohol abuse/eating disorders and stress by job situation (n = 1,031)

|  | |  |  | Job situation |  | *P* | φ |
| --- | --- | --- | --- | --- | --- | --- | --- |
| Variable | |  | employed (n = 591) | unemployed (n = 199) | retired  (n = 241) |  |  |
| Depression | % (n) | | 29.1% (172) | 39.7% (79) | 17.0% (41) | <0.001 | 0.165 |
| Anxiety | % (n) | | 17.4% (103) | 19.1% (38) | 10.4% (25) | 0.019 | 0.088 |
| Insomnia | % (n) | | 13.7% (81) | 21.1% (42) | 11.2% (27) | 0.009 | 0.095 |
| Alcohol Abuse | % (n) | | 20.3% (120) | 17.1% (34) | 12.9% (31) | 0.038 | 0.080 |
| Eating Disorder | % (n) | | 27.2% (161) | 29.6% (59) | 20.3% (49) | 0.053 | 0.075 |
| Moderate/High Stress | % (n) | | 67.9% (401) | 81.4% (162) | 43.6% (105) | <0.001 | 0.268 |

**Supplementary Table 6** Proportion of participants exceeding the cut-off scores for moderate depression/anxiety/insomnia/alcohol abuse/eating disorders and stress by education (n = 1,031)

|  | |  |  |  | Education |  |  | *P* | φ |
| --- | --- | --- | --- | --- | --- | --- | --- | --- | --- |
| Variable | |  | no/secondary  school  (n = 221) | Apprentice- ship  (n = 350) | Vocational  secondary  school (n= 169) | High  School   (n = 169) | University    (n = 122) |  |  |
| Depression | % (n) | | 33.9% (75) | 29.4% (103) | 26.0%  (44) | 30.2% (51) | 15.6% (19) | 0.007 | 0.117 |
| Anxiety | % (n) | | 21.3% (47) | 16.3% (57) | 14.2% (24) | 14.2% (24) | 11.5% (14) | 0.125 | 0.084 |
| Insomnia | % (n) | | 20.4% (45) | 15.7% (55) | 14.8% (25) | 10.7% (18) | 5.7% (7) | 0.003 | 0.125 |
| Alcohol Abuse | % (n) | | 21.7% (48) | 16.3% (57) | 15.4% (26) | 19.5% (33) | 17.2% (21) | 0.423 | 0.061 |
| Eating Disorder | % (n) | | 32.1% (71) | 26.3% (92) | 23.7% (40) | 27.2% (46) | 16.4% (20) | 0.029 | 0.102 |
| Moderate/High Stress | % (n) | | 75.6% (167) | 62.0% (217) | 63.9% (108) | 63.9% (108) | 55.7% (68) | 0.002 | 0.128 |

**Supplementary Table 7** Proportion of participants exceeding the cut-off scores for moderate depression/anxiety/
insomnia/alcohol abuse/eating disorders and stress by migration (n = 1,031)

|  | |  | Migration Background | | *P* | φ |
| --- | --- | --- | --- | --- | --- | --- |
| Variable | |  | yes (n= 140) | no  (n = 891) |  |  |
| Depression | % (n) | | 35.0% (49) | 27.3% (648) | 0.059 | -0.059 |
| Anxiety | % (n) | | 17.1% (24) | 15.9% (142) | 0.718 | -0.011 |
| Insomnia | % (n) | | 17.1% (24) | 14.1% (126) | 0.349 | -0.029 |
| Alcohol Abuse | % (n) | | 25.7% (36) | 16.7% (149) | 0.010 | -0.080 |
| Eating Disorder | % (n) | | 35.7% (50) | 24.6% (219) | 0.005 | -0.087 |
| Moderate/High Stress | % (n) | | 78.6% (140) | 62.6% (558) | <0.001 | 0.114 |

**Supplementary Table 8** Proportion of participants exceeding the cut-off scores for moderate depression/anxiety/
insomnia/alcohol abuse/eating disorders and stress by partnership status (n = 1,031)

| Variable | |  | Single (n= 322) | Partnership (n = 709) | *P* | φ |
| --- | --- | --- | --- | --- | --- | --- |
| Depression | % (n) | | 36.0% (115) | 24.8% (176) | <0.001 | -0.115 |
| Anxiety | % (n) | | 20.2% (65) | 14.2% (101) | 0.016 | -0.075 |
| Insomnia | % (n) | | 18.6% (60) | 12.7% (90) | 0.012 | -0.078 |
| Alcohol Abuse | % (n) | | 18.9% (61) | 17.5% (124) | 0.573 | -0.018 |
| Eating Disorder | % (n) | | 29.2% (94) | 24.7% (175) | 0.126 | -0.048 |
| Moderate/High Stress | % (n) | | 73.0% (235) | 61.1% (433) | <0.001 | -0.116 |
